# Mediation of Systemic Inflammation Response Index in the Association of Healthy Eating Index-2020 in Patientis with Periodontitis 

**DOI:** 10.3290/j.ohpd.c_1946

**Published:** 2025-04-15

**Authors:** Jiajia Yang, Liqin Gong, Xinchen Zhu, Yueyan Wang, Chong Li

**Affiliations:** a Jiajia Yang Attending Physician, Prosthodontics Department of Wuxi Stomatology Hospital, Wuxi City, JiangSu Province, China. Conceptualisation, formal analysis, funding acquisition, methodology, project administration, wrote the original draft.; b Liqin Gong Associate Chief Physician, Prosthodontics Department of Wuxi Stomatology Hospital, Wuxi City, JiangSu Province, China. Conceptualisation, formal analysis, investigation, wrote the original draft.; c Xinchen Zhu Attending Physician, Prosthodontics Department of Wuxi Stomatology Hospital, Wuxi City, JiangSu Province, China. Data curation, resources, software, wrote the original draft.; d Yueyan Wang Chief Physician, Prosthodontics Department of Wuxi Stomatology Hospital, Wuxi City, JiangSu Province, China. Data curation, validation, reviewed and edited the manuscript.; e Chong Li Attending Physician, Prosthodontics Department of Wuxi Stomatology Hospital, Wuxi City, JiangSu Province, China. Supervision, visualisation, project administration, reviewed and edited the manuscript.

**Keywords:** HEI-2020, mediation analysis, periodontitis, SIRI.

## Abstract

**Purpose:**

To investigate the function of the Systemic Inflammation Response Index (SIRI) in the association of Healthy Eating Index (HEI) 2020 in patients with periodontitis risk.

**Materials and Methods:**

This study utilised data from the National Health and Nutrition Examination Survey (NHANES) from 2009 to 2014, including participants’s oral examination results, dietary records, and levels of inflammatory markers. The study employed HEI-2020 as the independent variable and periodontitis as the dependent variable, using weighted logistic regression analysis to examine the association between HEI-2020 and periodontitis. Additionally, restricted cubic splines (RCS) were employed to further explore the non-linear association between the two. Mediation analysis was conducted to investigate the role of SIRI in the association between HEI-2020 and periodontitis.

**Results:**

3829 (34.5%) of the 9569 patients were diagnosed with periodontitis. In the weighted logistics regression model, HEI-2020 and the risk of periodontitis showed a statistically significant negative association (OR: 0.99, 95% CI: 0.98–1.00, p < 0.001). The findings of the RCS curve showed a linear correlation (p_non-linear_=0.684) between the risk of periodontitis and HEI-2020. With a mediation proportion of 9.82% (p < 0.001), the findings of the mediation study indicated that SIRI partially mediated the relationship between HEI-2020 and periodontitis.

**Conclusion:**

HEI-2020 and periodontitis risk are statistically significantly negatively correlated, and SIRI is a major mediating factor in this relationship. The study results may help clinicians better understand how a healthy diet impacts the risk of periodontal disease and identify the mediating role of SIRI in this association. This knowledge can guide personalised dietary and inflammation management strategies, enhancing oral and overall health by preventing and managing periodontal issues effectively.

Periodontitis is a common chronic inflammatory disease, the pathogenesis of which involves a complex interplay between periodontal pathogens, host inflammation and immune responses, and other environmental risk factors.^
40
^ This condition leads to irreversible damage to periodontal supporting tissues, such as the gums, alveolar bone, cementum, and periodontal ligaments. If not treated promptly, it may result in tooth loss and impair the patient’s chewing function and quality of life.^
10,35
^ Additionally, periodontitis may disseminate inflammatory mediators throughout the body via the bloodstream and the chewing process, increasing the risk of systemic inflammatory diseases.^
3
^ In addition to having an impact on personal health, periodontitis is a major global public health concern. As to the most recent data, the number of people suffering from periodontal disease grew from 792 million in 1990 to 1.07 billion in 2021, with 89.6 million new cases occurring; in contrast, this was 70.6 million in 1990.^
10
^ Globally, periodontitis (including tooth loss) is estimated to cost $186 billion in direct treatment expenditures and $142 billion in lost productivity.^
31
^ Therefore, it is particularly important to implement effective prevention and intervention measures to mitigate the global economic burden caused by periodontitis.

An increasing amount of evidence indicates that nutrition is a major modifiable component in periodontitis and that a healthy diet low in carbohydrates, rich in vitamin C, and rich in fiber alleviates periodontitis, while consuming an unhealthy diet high in sugar, low in vitamin C, and low in fiber leads to an increased risk of periodontitis.^
22,23,29
^ Nevertheless, the majority of earlier research has focused on the function of a single nutrient or a specific set of nutrients, failing to sufficiently take into account the interactions between different nutrient combinations in the diet.^
44
^ Thus, to investigate the relationship between food types and periodontitis, we need to concentrate more on comprehensive, healthy eating patterns. The Healthy Eating Index-2020 (HEI-2020) is a tool to measure overall dietary quality developed to evaluate how well Americans’ eating habits correspond with the most recent Dietary Guidelines for Americans, 2020–2025.^
37
^ Higher HEI-2015 scores are not as strongly linked to a decreased risk of developing periodontitis (OR: 0.69, 95% CI: 0.49–0.97, p = 0.033),^
19
^ but the HEI-2020 is more significant for research evaluating and improving the health effect of dietary habits, since it was able to incorporate the most current modifications in dietary guidelines (https://epi.grants.cancer.gov/hei/comparing.html). By selecting foods and drinks that fit an individual’s budget and tastes while adhering to the most recent dietary recommendations, it may be possible to maintain health and prevent periodontitis by investigating the relationship between the most recent HEI-2020 Index and the risk of periodontitis.

The Systemic Inflammation Response Index (SIRI) is a comprehensive inflammatory marker that includes information on neutrophils, monocytes, and lymphocytes. It offers a more comprehensive assessment compared to indicators based on a single or dual type of immune inflammatory cells.^
12,48
^ According to Ren et al,^
34
^ a correlation exists between higher SIRI levels and a higher risk of periodontitis (OR: 1.18, 95% CI: 1.01–1.39, p = 0.04.^
34
^ A systematic review of research indicated that dietary patterns could influence immune-mediated inflammatory responses and identified a link between inadequate diet and an elevated risk of periodontal disease.^
27
^ For instance, Wang et al^
43
^ discovered that a high HEI-2015 score was linked to a lower level of systemic inflammation (OR: 0.76, 95% CI: 0.69–0.84, p ≤ 0.001). Based on these findings, we hypothesise that inflammation may play a mediating role in the relationship between diet and the risk of periodontitis. This study investigated whether SIRI mediates the correlation between HEI-2020 and periodontitis and evaluated this relationship using data from the National Health and Nutrition Examination Survey (NHANES) database. To provide a theoretical foundation for the development of comprehensive dietary and lifestyle treatments to prevent periodontal disease, the purpose of this study was to obtain a deeper understanding of the possible mechanisms relating to food, inflammation, and periodontitis.

## Materials and Methods

### Ethics Approval and Consent to Participate

Before data from this study were included in the NHANES public database, all participants signed informed consent forms in accordance with the principles outlined in the Declaration of Helsinki. These were reviewed and approved by the NCHS Ethical Review Board.

### Data Source and Study Population

Every two years, the National Center for Health Statistics (NCHS) in the United States conducts the NHANES national survey, which evaluates the population’s health and nutrition. The NCHS Institutional Review Board approved the research protocol, and each participant has given their informed consent. Data from the NHANES survey are accessible to the public online at https://www.cdc.gov/nchs/nhanes/index.htm.

Data from a total of 30,468 individuals from three cycles of the 2009–2014 NHANES survey were used. The final sample consisted of 9569 people, after missing or unavailable data on inflammation (n = 393), diet (n = 2540), and periodontitis (n = 16,397), as well as other missing covariate data (n = 1569), were excluded. Figure 1 depicts the procedure used to choose the study population.

### Exposure Variable: HEI-2020

Two 24-h dietary recall interviews were used to gather NHANES dietary intake data. To determine each participant’s daily HEI components, the study calculated two dietary intake recall sessions.^
45
^ The researchers utilised the R language package “Dietaryindex”, created by Zhan et al to compute the HEI-2020.^
46
^ Using this technique, nutritional consumption data is normalised to provide a dietary pattern index.

Thirteen components make up the HEI Index: nine adequate components (total fruits, whole fruits, total vegetables, greens and beans, whole grains, dairy, total protein foods, seafood and plant proteins, fatty acids), and four moderate ingredients (refined grains, sodium, added sugars, saturated fats).^
15
^ Referring to the scoring rules of Reedy et al,^
33
^ total fruits, whole fruits, total vegetables, greens and beans, total protein foods, and seafood and plant proteins are scored on a scale of 0-5, while the other seven components are scored on a scale of 0-10. The HEI-2020 score ranges from 0 to 100, with higher scores reflecting higher dietary quality.

### Outcome Variable: Periodontitis

An examiner from the NHANES mobile examination facility performed periodontal exams. To determine the periodontal state of eligible survey respondents, the examiner conducted a full-mouth periodontal examination. For a total of 168 sites per dentition,^
7
^ the examiner examined gingival recession (GR) and probing depth (PD) at six sites per tooth (buccal, mesiobuccal, distobuccal, mesiolingual, lingual, and distolingual). The severity of periodontitis was then assessed by computing the difference between GR and PD, or Clinical Attachment Loss (CAL).^
6
^


The diagnosis of periodontitis is determined based on the consensus recommendations of the Centers for Disease Control and Prevention (CDC) and the American Academy of Periodontology (AAP) epidemiological studies.^
5,28
^ Periodontitis is divided into three categories: mild, moderate, and severe periodontitis. Mild periodontitis is defined as CAL ≥ 3 mm in ≥ 2 interproximal sites and PD ≥ 4 mm in ≥ 2 interproximal sites (not on the same tooth) or PD ≥ 5 mm in 1 site. Moderate periodontitis is defined as CAL ≥ 4 mm in ≥ 2 interproximal sites (not on the same tooth) or PD ≥ 5 mm in ≥ 2 interproximal sites (not on the same tooth). Severe periodontitis is defined as CAL ≥ 6 mm in ≥ 2 interproximal sites (not on the same tooth) and PD ≥ 5 mm in ≥ 1 interproximal site.^
8
^ In this study, the participants were divided into two groups based on the presence of periodontitis: non-periodontitis vs periodontitis (including mild periodontitis and moderate/severe periodontitis).

### Mediator: SIRI

The MEC automated hematology analyser (Beckman Coulter MAXM, Beckman Coulter; Brea, CA, USA) was used to examine the blood samples that were obtained. SIRI was computed using the following formula: SIRI = (neutrophil count × monocyte count) / lymphocyte count.^
4
^


### Covariates

The covariates were gender, age, ethnicity, body mass index (BMI), ratio of family income to poverty (poverty:income ration, PIR), alcohol consumption, and physical activity.

Ethnic groups included Mexican Americans, other Hispanics, non-Hispanic blacks and whites, and other ethnicities. BMI categories were as follows: underweight/healthy weight: < 25 kg/m^
2
^, overweight: 25–30 kg/m^
2
^, or obese: > 30 kg/m^
2
^.^
12
^ PIR was used to measure economic status and was divided into three categories: low income: < 1.3; middle income: 1.3–3.5; and high income: ≥ 3.5.^
18
^ Alcohol consumption was defined as > 12 drinks per year, with a “drink” being 355 ml beer, one 148-ml glass of wine, or 44 ml of hard liquor. Physical activity was divided into three groups: none, moderate, or intense.^
4
^


### Statistical Analysis Methods

R was used for all statistical analyses (V4.3.3). Using the “tableone” package (https://github.com/kaz-yos/tableone), the baseline table was created, classifying individuals according to their periodontitis status and general demographic characteristics. The sample size and percentage (n[%]) for categorical variables were displayed, whereas the mean (±SD) was used for continuous variables. Using the “survey” package (http://r-survey.r-forge.r-project.org/survey/), a weighted logistic regression model was built to evaluate the relationship between HEI-2020 (continuous and tertiles) and periodontitis. The findings were expressed as odds ratios (OR) and their 95% confidence intervals (CI). Three models were constructed: a crude model that was not modified; model 1 that was adjusted for age, gender, and ethnicity; model 2 that was adjusted for BMI, alcohol drinking, PIR, and physical activity. Restricted cubic splines (RCS) were utilised in the regression model to further investigate the association between HEI-2020 and periodontitis after adjusting for all confounding variables.^
47
^ A regression model was built using the “mediation” package (https://imai.princeton.edu/projects/mechanisms.html) to investigate any potential mediating role that SIRI may have on the relationship between HEI-2020 and periodontitis. Statistical significance was set at p < 0.05.

## Results

### Baseline Characteristics

3829 (34.5%) of the 9569 individuals in this study, who were drawn from the NHANES database between 2009 and 2014, had periodontitis. In Table 1, baseline characteristics are displayed. The average age of the total study population was 52.8 ± 14.0 years, with 4920 (51.6%) females and a majority of non-Hispanic Whites (71.7%). Patients with periodontitis and those without it differed statistically significantly in terms of age, gender, ethnicity, PIR, SIRI, and HEI-2020 (p < 0.05). Patients with periodontitis were older (54.9 vs 51.6) than those without, most were men (58.1% vs. 43.3%), a larger share had a middle income (41.0% vs 30.9%), and a higher SIRI level (1.4 vs 1.2). Furthermore, patients with periodontitis had a stastitically significantly lower HEI-2020 than patients without the condition (51.8 vs 53.6, p < 0.001).

**Table 1 table1:** Characteristics of NHANES Participants between 2009–2014

Characters	Total	Non-periodontitis	Periodontitis	p-value
Overall	9569	5737 (65.5)	3829 (34.5)	
Age	52.76 (13.98)	51.63 (14.13)	54.92 (13.42)	<0.001
**Gender**				<0.001
Male	4649 (48.4)	2418 (43.3)	2231 (58.1)	
Female	4920 (51.6)	3319 (56.7)	1601 (41.9)	
**Ethnicity**				<0.001
Mexican American	1218 (7.1)	564 (5.4)	654 (10.5)	
Other Hispanic	840 (4.7)	484 (4.3)	356 (5.5)	
Non-Hispanic White	4682 (71.7)	3115 (76.0)	1567 (63.4)	
Non-Hispanic Black	1915 (10.1)	999 (8.2)	916 (13.7)	
Other	914 (6.4)	575 (6.1)	339 (6.8)	
**BMI (kg/m** ^ 2 ^ **)**				0.258
<25	2443 (26.5)	1510 (27.3)	933 (24.9)	
25–30	3275 (34.9)	1948 (34.5)	1327 (35.6)	
≥30	3851 (38.6)	2279 (38.2)	1572 (39.5)	
**Alcohol consumption**			0.689
No	2584 (22.1)	1577 (22.3)	1007 (21.6)	
Yes	6985 (77.9)	4160 (77.7)	2825 (78.4)	
**Physical activities**			0.372
None	2958 (36.0)	1782 (36.4)	1176 (35.2)	
Moderate	3313 (35.1)	1983 (35.3)	1330 (34.7)	
Intense	3298 (28.9)	1972 (28.2)	1326 (30.2)	
**PIR**				<0.001
Low	2846 (19.5)	1537 (16.4)	1309 (25.3)	
Medium	3475 (34.4)	1948 (30.9)	1527 (41.0)	
High	3248 (46.1)	2252 (52.7)	996 (33.7)	
SIRI	1.27 (0.88)	1.23 (0.85)	1.35 (0.92)	0.001
HEI-2020	52.99 (12.03)	53.61 (12.07)	51.81 (11.87)	<0.001
Note: Categorical variables are expressed as n(%) and continuous variables are expressed as mean(sd); n is unweighted, and n(%), mean and SD are weight-adjusted. BMI: body mass index; PIR: poverty income ratio; SIRI: system inflammation response index; HEI: healthy eating index.

### Regression Analysis

To investigate the link between HEI-2020 (continuous and tertiles) and periodontitis, a weighted logistic regression model was constructed. In the adjusted model 2, HEI-2020 was statistically significantly adversely linked with the risk of periodontitis (OR: 0.99, 95% CI: 0.98–1.00, p < 0.001), as shown in Table 2 when HEI-2020 was treated as a continuous variable. When HEI-2020 was further stratified into weighted tertiles, all models exhibited a statistically significant trend with p_trend_<0.01, suggesting that the risk of periodontitis decreased as HEI-2020 tertiles increased.

**Table 2 table2:** Associations between HEI-2020 and periodontitis

Participants	Crude	Model 1 OR (95% CI)	Model 2
HEI-2020 (continuous)	0.99 (0.98–0.99) ***	0.99 (0.98–0.99) ***	0.99 (0.98–1.00) **
HEI-2020 (categorical)
Q1 (≤46.93)	Ref.	Ref.	Ref.
Q2 (46.93-57.83)	0.89 (0.77–1.03)	0.85 (0.72–0.99) *	0.88 (0.75–1.03)
Q3 (>57.83)	0.70 (0.59–0.82) ***	0.67 (0.57–0.80) ***	0.74 (0.62–0.90) **
p_trend_	<0.001	<0.001	0.001
Note: Crude unadjusted; model 1 adjusted for age, ethnicity, and gender; model 2 adjusted for age, ethnicity, gender, BMI, smoking, alcohol consumption, PIR, and physical activity. OR: Odds ratio; CI: confidence interval. *p < 0.05, ** p < 0.01, *** p < 0.001.

### RCS Analysis

**Fig 2 Fig2:**
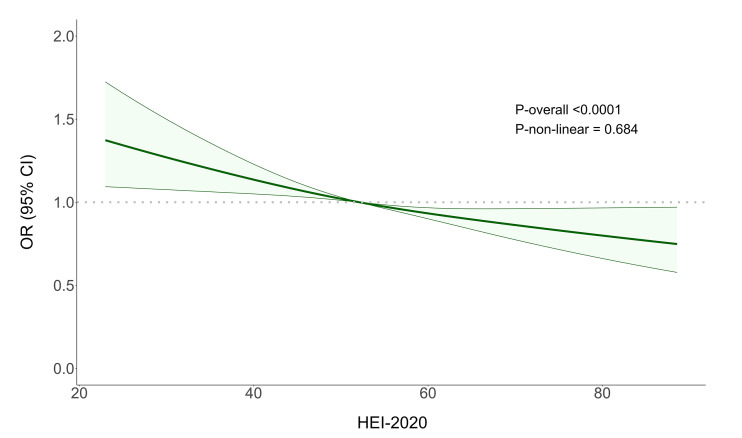
OR of HEI-2020 and periodontitis adjusted by covariates, NHANES 2009–2014. RCS line is adjusted for age, ethnicity, gender, BMI, alcohol drinking, physical activities, PIR. The OR is represented by the green line and the shaded area represents 95% CI. OR: odds ratio; CI: confidence interval.

Figure 2 illustrates the non-linear relationship between HEI-2020 and the risk of periodontitis after adjusting for all confounders. The results indicated a statistically significant overall trend between HEI-2020 and the risk of periodontitis (p_overall_<0.0001), meaning that as HEI-2020 increased, the risk of periodontitis decreased statistically significantly. The non-linear association between HEI-2020 and periodontitis was not statistically significant (p_non-linear_=0.684).

### Mediation Analysis

**Fig 3 fig3:**
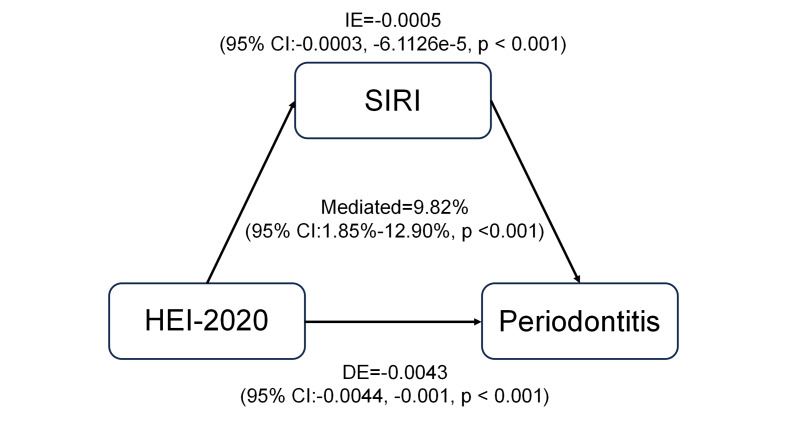
Results of SIRI-mediated HEI-2020 and periodontitis.

This study evaluated the possible mediating role of SIRI on the relationship between HEI-2020 and periodontitis to investigate the possible processes underpinning the correlation between HEI-2020 and the risk of periodontitis. As shown in Fig 3, the findings demonstrated that HEI-2020 had direct and indirect impacts on periodontitis of -0.0043 and -0.0005, respectively (p < 0.001), suggesting that a higher HEI-2020 level was linked to a lower risk of periodontitis. The relationship between HEI-2020 and the risk of periodontitis was statistically significantly mediated by SIRI, with a mediation proportion of 9.9% (p < 0.001).

## Discussion

This study investigated the association between HEI-2020 and periodontitis, as well as the possible mediating function of SIRI in this relationship, using data from the NHANES database spanning the years 2009 to 2014. The findings showed a statistically significant negative linear correlation between the risk of periodontitis and HEI-2020, with SIRI playing a statistically significant mediating role. The mediation effect accounted for 9.8% of the relationship.

The results of this study showed a strong negative relationship between the risk of periodontitis and HEI-2020. In keeping with our results, Li et al^
20
^ used the NHANES database to investigate the relationship between HEI-2015 and periodontitis. They discovered that those with greater HEI-2015 had a decreased probability of developing periodontitis (OR: 0.69, 95% CI: 0.55-0.86, p = 0.001).^
20
^ They also noted that among the components of HEI-2015, total fruits, total vegetables, whole grains, fatty acids, and seafood and plant proteins were associated with low risk of periodontitis prevalence.^
20
^ This may be related to oxidative stress and host immunity. A review article supports that view, and it suggests that fruits, vegetables, and whole grains have antioxidant properties that can improve periodontal health.^
26
^ Shang et al^
38
^ proposed that periodontal infections induce neutrophil aggregation in periodontal tissues, which increases oxidative stress and reactive oxygen species generation, hence aggravating periodontal tissue destruction.^
38
^ Omega-3 polyunsaturated fatty acid dietary supplements are considered effective in modulating host immune responses and reducing pro-inflammatory cytokine production, thereby improving periodontal clinical parameters.^
2,16
^ Additionally, a clinical study has shown that vegetarians typically exhibit lower levels of inflammation and less periodontal damage, which may be associated with their healthier lifestyle.^
39
^ Therefore, to promote periodontal health, the present authors recommend focusing on a healthy diet, supplementing essential nutrients, enhancing immune system function, and generally maintaining a healthy lifestyle, which includes regular exercise, stress management, and good oral hygiene practices to prevent and manage periodontitis.

This study found a statistically significant linear relationship between HEI-2020 and the risk of periodontitis. In contrast, the findings of Li et al^
19
^ showed a non-linear relationship between HEI-2015 and periodontitis. The reasons for the contradictory results of the two studies may include the following. First, the studies used different versions of HEI. We used the latest version of HEI-2020, which reflects the updated dietary guidelines changes in the Dietary Guidelines for Americans, 2020-2025. Due to emerging needs and changes, dietary guidelines are updated every 5 years; thus, to maintain consistency with the latest Dietary Guidelines, HEI is also continuously updated and developed.^
32
^ The greatest change in the 2020 edition compared to the 2015 edition of the Dietary Guidelines is the first-time provision of healthy eating pattern guidance according to life stage (from birth to old age, including pregnant or lactating women) (https://www.dietaryguidelines.gov/resources/2020-2025-dietary-guidelines-online-materials). Second, the sample sizes and periods of the included populations differed, and during this period, the overall dietary quality of the U.S. population has steadily improved, including increases in whole fruits, whole grains, and polyunsaturated fatty acid intake.^
41
^


With a 9.8% mediation ratio, the mediation study demonstrated that SIRI strongly mediated the association between HEI-2020 and the probability of acquiring periodontitis. This emphasises that there is a direct correlation between periodontitis and diet quality, and that inflammation plays a pivotal role in this process. Prior research has indicated a connection between diet and inflammatory biomarkers, with calorie restriction inhibiting the inflammatory response and lowering inflammatory mediators in gingival sulcus fluid, including interleukin-6, tumor necrosis factor-α, and plasma C-reactive protein.^
30
^ According to Li et al,^
17
^ a pro-inflammatory diet is linked to a higher chance of developing periodontitis (OR: 1.53, 95% CI: 1.33-1.77, p < 0.05). This further supports the idea that diet-mediated inflammation regulation may be a key pathway linking the HEI-2020 to periodontal health. Negative relationships between HEI-2015 and inflammatory markers, including C-reactive protein, white blood cells, and neutrophils, have also been reported.^
24
^ Drawing from this, we postulated that diet may reduce inflammation levels through several mechanisms, including inhibiting the expression of the nuclear transcription factor NF-κB, regulating host immune responses, altering the composition of oral microbiota, and suppressing NLRP3 inflammasome activation.^
13,14,36
^ Inflammation could be a major factor in the pathophysiology of periodontitis. Numerous investigations have proven the link between inflammatory biomarkers and periodontitis.^
1,25
^ According to Gomes-Filho et al,^
11
^ individuals with chronic periodontitis had a greater likelihood of having higher levels of C-reactive protein (OR: 2.16, 95% CI: 1.2-3.9, p = 0.011). An inflammatory diet statistically significantly influenced the correlation between systemic inflammation and periodontitis (p < 0.01), according to a study examining the relationship between dietary inflammatory indices and periodontitis.^
21
^


This study not only supports the central role of inflammation in the pathological process of periodontitis but also deepens our understanding of the complex interactions between diet, inflammation, and periodontitis through the novel indicator SIRI. As an effective marker of systemic inflammation, this study is the first to explore the mediating role of SIRI in the relationship between HEI-2020 and the risk of periodontitis, providing new strategies for optimising dietary patterns to regulate systemic inflammation and prevent/manage periodontitis. These findings enhance our understanding of the mechanisms underlying periodontitis and provide a scientific basis for the design of future interventions. Furthermore, to ensure that dietary changes can be implemented by dental professionals and patients, the present authors recommend the following measures. First, dental practitioners can educate patients on the importance of a healthy diet for periodontal health, thereby increasing their awareness of HEI-2020. Second, personalised dietary plans can be developed, taking into account patients’ budgets and preferences, to promote the establishment of healthy eating habits. Additionally, dental clinics can collaborate with nutritionists to provide patients with professional dietary consultations and support. Through these measures, patient adherence to healthy eating can be enhanced, thereby reducing the risk of periodontitis.

This study has some limitations. First, due to its cross-sectional design, it was not possible to determine a causal relationship and directionality between HEI-2020 and periodontitis. This design limits the ability to infer temporal sequence and causality. Second, the study data rely on self-reports from participants, which may introduce recall bias and reporting inaccuracies. Moreover, although we adjusted for several potential confounding factors, there may still be unidentified or uncontrolled confounders, such as oral hygiene practices, brushing techniques, and frequency, that could affect the relationship between HEI-2020 and periodontitis. Additionally, while our sample derives from the nationally representative NHANES database, the selection and time period limitations may impact the generalisability of the results. The sample may not fully represent all populations, particularly those from different cultural, geographic, and socioeconomic backgrounds. Lastly, this study did not delve into how dietary quality interacts with oral microbiota and other factors influencing the risk of periodontitis, which is an important knowledge gap that future research needs to address.

## Conclusion

The results indicated a negative linear correlation between HEI-2020 and the risk of periodontitis, with SIRI statistically significantly mediating this association. A healthy dietary pattern is beneficial for preventing periodontitis, and it is recommended that dental professionals incorporate oral nutrition into routine dental care to improve the prognosis of periodontal disease patients and reduce costly treatment measures. Furthermore, we suggest that when HEI-2020 interventions are needed in the prevention or treatment of periodontitis, a focus should be placed on systemic inflammatory biomarkers. Future research should further conduct longitudinal studies to confirm the causal relationship and dynamic changes between HEI-2020 and periodontitis.

## References

**Fig 1 Fig1:** Screening flowchart of the study population.
